# Barium Titanate/Gadolinium Ferrite: A New Material Composite to Store Energy

**DOI:** 10.3390/nano13131955

**Published:** 2023-06-27

**Authors:** Clara Baivier, Imen Hammami, Ratiba Benzerga, Manuel P. F. Graça, Luís C. Costa

**Affiliations:** 1 CNRS, IETR–UMR 6164, University of Rennes, 35000 Rennes, France; clara.baivier@etudiant.univ-rennes.fr (C.B.); ratiba.benzerga@univ-rennes.fr (R.B.); 2 I3N and Department of Physics, University of Aveiro, 3810-193 Aveiro, Portugal; imenhammami@ua.pt (I.H.); mpfg@ua.pt (M.P.F.G.)

**Keywords:** nanocomposites, dielectric properties, energy storage, impedance spectroscopy

## Abstract

This work investigates the dielectric properties of barium titanate/gadolinium ferrite ceramic composites, with different concentrations of each material. Our objective was to increase the storage ability of this material, finding a compromise between high permittivity and low dielectric losses. A two-step sintering procedure was used in the preparation of the composites to attain the desired results. Their morphological, structural and electrical properties were tested using scanning electron microscopy, X-Ray powder diffraction and impedance spectroscopy, respectively. Dielectric characterizations were performed on the frequency band of 100 Hz–1 MHz and for different temperatures (180–380 K). The best compromise between barium titanate and gadolinium ferrite in the composition was calculated in order to obtain a potential material for electrical energy storage. The sample with 25% gadolinium ferrite presented the best results. The dielectric constant reached values of the order of 2000, at 1 kHz and 340 K. It was also important not to have very high losses, and this was confirmed by the calculated loss tangent.

## 1. Introduction

The demand for high-performance, environmentally friendly and low-cost components is responsible for growth in the study of composite materials. It is known that one of the most significant motivations for studying these materials is their physical and chemical characteristics, which are largely enhanced when compared to those of traditional ceramic materials.

In the electronics industry, it is important to minimize the size of components. In particular, for energy storage, capacitors are commonly used. The choice of the capacitor technology depends on the application, conditioning energy density and costs. However, applications require materials with a high dielectric constant and low losses. In the case of ceramics, an optimized material can be achieved, thanks to the infinity of possible chemical compositions to be realized by cationic substitutions or by assembling different materials [1,2].

Barium titanate, BaTiO_3_, (BTO) is a ferroelectric material known for its dielectric properties: on account of its high permittivity and low dielectric loss, this material is particularly used for the manufacture of electronic components, such as multilayer ceramic capacitors [3]. Due to its perovskite (ABO_3_) structure, BTO has the advantage of being able to be subjected to numerous cationic substitutions in its lattice, thus modifying its physico-chemical properties. It can be synthetized using several methods, such as solid-state reactions, sol-gel and chemical vapor deposition, and the obtained physical properties can be tuned, changing the procedure parameters and also its composition. In the same way, this compound offers the possibility to realize new microstructures with multiferroic properties by integrating distinct materials into its matrix, such as ferrites [4].

Ferrite-based materials are magnetic oxides, whose main magnetic element is iron. Their excellent magneto-dielectric properties, such as high electrical resistivity and low manufacturing costs, make them a material of choice for many electronic applications. In particular, rare-earth ferrites exhibit excellent electronic and magnetic properties, such as high coercivity, magnetic anisotropy and low losses [5,6,7]. Different methods can be used to obtain these materials, such as the solid-state route, sol-gel and chemical co-precipitation [8].

Changing their phase composition and morphology by the addition of new components such as ferrites is one of the most important research topics currently, due to the possibility of obtaining new materials with enhanced properties compared to those of the original ones.

Rare-earth compounds have been sintesized with particularly interesting electric and magnetic properties [9,10], and this is a good base for developing new materials for electronic applications.

In this work, we focused on the addition of rare-earth iron garnet (REIG) ferrites, such as gadolinium iron garnets (GIGs), in a barium titanate (BTO) matrix. Indeed, REIGs have good dielectric properties, with a high dielectric constant and low dielectric losses, which make them promising candidates for electrical devices [11,12,13,14]. One of the particularities of iron garnets is the fact that all their cations are trivalent, which gives them a high resistivity. Dielectric losses are linked with the simultaneous presence of Fe^2+^ and Fe^3+^ ions located in close crystallographic sites [3]. Other ferrites have already been studied with promising results, including spinel ferrites and hexaferrites [15,16].

In order to observe the influence of the addition of GIGs in the BTO matrix on the microstructural and dielectric properties, we focused on the elaboration of ceramic BTO + GIG composites by modifying the added proportion of GIGs. This was motivated by their low cost and the use of environmentally friendly materials, avoiding composite materials that contain Pb. Our main objective was to obtain a material with the highest dielectric constant possible, while maintaining the dielectric losses as low as possible.

## 2. Materials and Methods

BaTiO_3_ (BTO) and Gd_3_Fe_5_O_12_ (GIG) powders as well as their composites were synthesized by solid-state reaction and subjected to a two-step sintering process [17], following the formula: (100-x) BTO + (x) GIG (x = 0, 25, 50, 75 and 100 wt%). We first prepared BTO and GIG powders. A mixture of BaCO_3_ (99+% pure, MaTeck, Jülich, Germany) and TiO_2_ (99.8% pure, Sigma-Aldrich, St. Louis, MO, USA) was prepared at room temperature. Several works present different examples of how to produce oxide compounds [18,19]. The powders were weighted in a 1:1 stoichiometric ratio to obtain BaTiO_3_. GIG was prepared using oxide powder of Fe_2_O_3_ (99% pure, Sigma-Aldrich, St. Louis, MO, USA) and gadolinium nitrate Gd(NO_3_)_3_.6 H_2_O according to the following equations:

(1) $$2\mathrm{Gd}{\left({\mathrm{NO}}_{3}\right)}_{3}\;\to {\mathrm{Gd}}_{2}O_{3}+6{\mathrm{NO}}_{2}+\frac{3}{2}O_{2}$$

(2) $$3{\mathrm{Gd}}_{2}O_{3}+5{\mathrm{Fe}}_{2}O_{3}\;\to 2{\mathrm{Gd}}_{3}{\mathrm{Fe}}_{5}O_{12}$$

The mixtures were each ball-milled in a planetary ball-milling system (Planetary Mill Pulverisette 5-FRITSCH) with 10 agate balls (with a 1 cm diameter) at 250 rpm for 1 h in a 200 mL agate vessel.

In order to define the temperature used in the heat treatments, the obtained powders were thermally analyzed by differential thermal analysis (DTA) and thermogravimetry analysis (TGA) with an STA7300 Thermal Analysis System (HITACHI, Tokyo, Japan). The samples were heated from room temperature up to 1200 °C with a heating rate of 10 °C/min, under N_2_ atmosphere.

The powders were calcined at 800 °C in the air for 12 h and then analyzed by X-ray diffraction. The calcined BTO and GIG powders were then mixed using ethanol and agate mortar as a medium at room temperature to produce three compositions: 25, 50 and 75 wt% of GIG in the BTO matrix.

For the pelletization, each composition was mixed with polyvinyl alcohol (PVA) as a binder and pressed into cylindrical pellets with a diameter of 7 mm and thickness of approximately 1.3 mm, under uniaxial pressure of 3 MPa. During the sintering process, the PVA binder was removed. Finally, the pellets were sintered at 1000 °C, in air, for 12 h. We also pressed BTO and GIG into pellets as a reference with the same sintering conditions.

**Figure 1:**
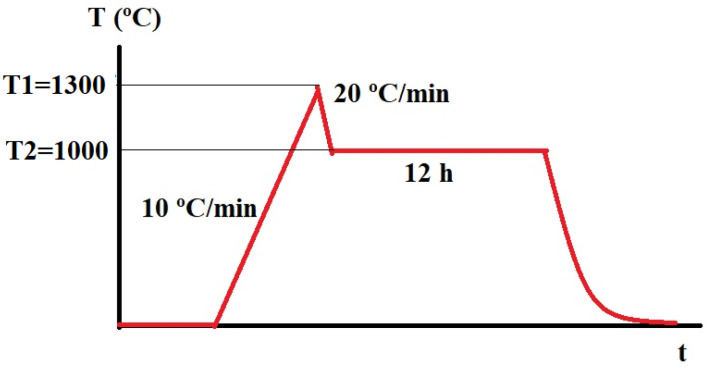
Thermal cycle for sintering of the samples.

Many studies have reported that it is possible to obtain dense ceramics for BTO and GIGs prepared by conventional sintering method and using a sintering temperature between 1000 °C and 1300 °C. However, it is also assumed that to obtain compounds that are mostly pure at 1000 °C, a two-step sintering (TSS) procedure is required [20]; therefore, we used this procedure. The samples were first heated at 10 °C/min to 1300 °C (T_1_), then cooled at 20 °C/min to 1000 °C (T_2_) and kept at 1000 °C for 12 h, as presented in Figure 1.

The crystallographic structure and the phase formation of our samples were studied by X-ray diffraction (XRD). We used an X-ray diffractometer PANalytical from AERIS, using Cu-K1 radiation (λ = 1.5406 Å) at 40 kV and 15.0 mA, in the 2θ angle range of 10°–70°, with step-size of 0.003°.

The surface and bulk morphology was evaluated by scanning electron microscopy (SEM) (Vega 3—TESCAN). The samples were coated with carbon before microscopic observation. 

For the electrical measurements, in the frequency range from 100 Hz to 1 MHz, pellets in disk shape were prepared and their opposite surfaces were painted with silver conducting paste. During the electrical measurements, the samples were maintained in a helium atmosphere to improve the heat transfer and eliminate the moisture. The measurements were performed using an Agilent 4294A impedance analyzer, as a function of the temperature (180–380 K) and frequency using the C_p_—R_p_ configuration. The temperature of the samples was regulated by an Oxford Research IT-C4 and monitored using Pt sensors.

## 3. Results

### 3.1. Thermo Analysis

**Figure 2:**
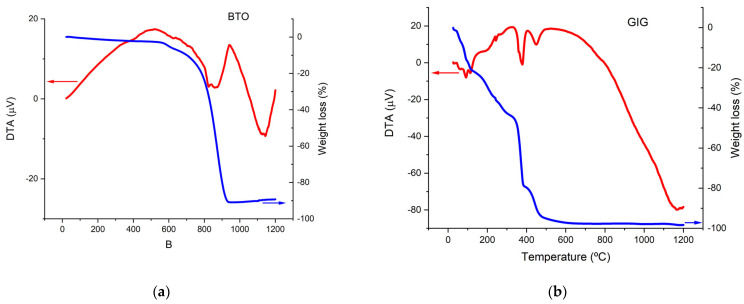
DTA (red) and TGA (blue) analyses for (**a**) BTO and (**b**) GIG powders.

To define the temperatures of the heating treatments, the pure GIG and pure BTO powders were analyzed by a differential thermal analysis and a thermogravimetry analysis. Figure 2 shows these results.

For the BTO, according to the DTA curves (Figure 2a), there are important changes around 900 °C. As can be seen by the TGA result, these changes are associated with a maximum weight loss of 16%. Around 600 °C, BaCO_3_ and TiO_2_, the precursors of BaTiO_3_, begin to react by releasing CO_2_ according to the following reaction:

BaCO_3_(s) + TiO_2_(s) → BaO(s) + CO_2_(g) + TiO_2_(s) → BaTiO_3_(s) (3)

The DTA peak in the temperature range of 800–950 °C is probably due to the formation of BaTiO_3_.

After 950 °C, a BaTiO_3_ single phase should be formed.

The representative DTA-TG curves of the GIG are given in Figure 2b. The TGA curve shows a maximum weight loss of 40%, which occurs before 600 °C. Between 50 °C and 150 °C, the compound loses around 10% of its mass; from the DTA result, we can see three endothermic peaks in this temperature range. These probably correspond to dehydration from losing its crystallization water.

Moreover, according to the literature, the decomposition of the nitrates of transition metals such as gadolinium occurs generally below 100 °C [21]. In the range of 500–600 °C, the remaining volatile products are lost, and the mass of the compound becomes practically constant.

According to these results and the literature, we selected 1000 °C and 1300 °C [22,23] as the temperatures for the heat treatments.

### 3.2. Structural Analysis

**Figure 3:**
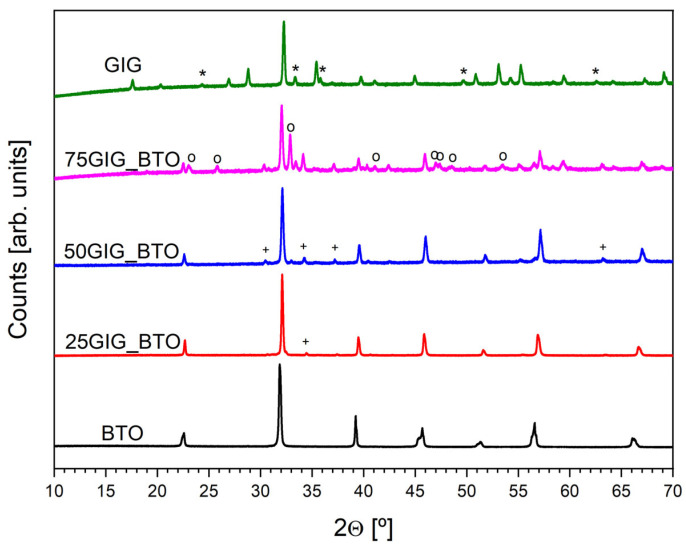
X-Ray diffraction pattern of all samples (* Fe_2_O_3_; o GdFeO_3_; + BaFe_12_O_19_).

Figure 3 shows the X-Ray diffraction results for the different samples.

The BTO sample showed a single-phase structure with the BaTiO_3_ crystal phase (matched by JCPDS ref. nº 04-016-3476). The X-ray pattern of the GIG sample shows the coexistence of two crystal phases: the expected Gd_3_Fe_5_O_12_ (ref. nº 04-002-7232) and Fe_2_O_3_ (ref. nº 01-089-0597). The sample with 25% GIG showed diffraction peaks associated with the BaTiO_3_ phase, but also some low-intensity peaks related to the BaFe_12_O_19_ phase, which are also present in the samples with 50 and 75% GIG. The intensity of these peaks increases with the increase in the GIG concentration. The X-ray pattern of the 75% sample shows the formation of a GdFeO_3_ perovskite structure.

### 3.3. Morphological Analysis

**Figure 4:**
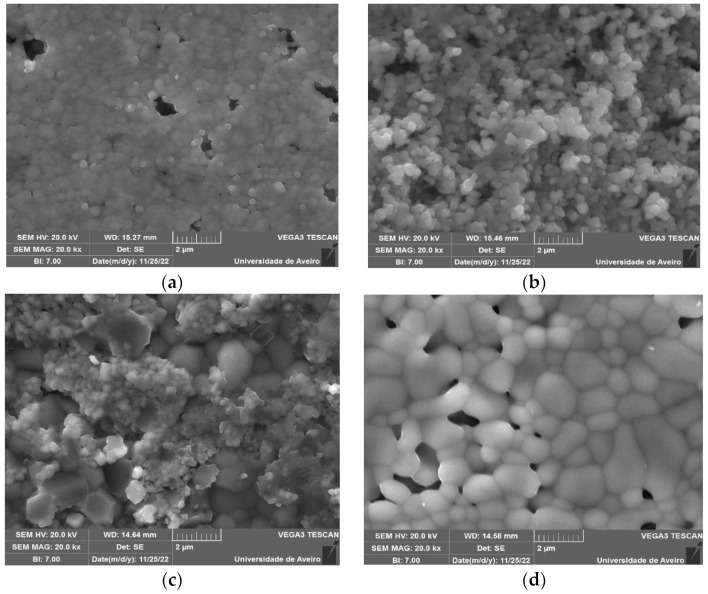
SEM images of different samples (magnitude of 20,000×) for (**a**) BTO (surface); (**b**) BTO (fracture); (**c**) 75GIG_BTO (surface); and (**d**) GIG (surface).

The scanning electron microscopy images are shown in Figure 4.

In the micrographic study of the BTO sample, represented by Figure 4a,b, the existence of grains with average dimensions of 200 nm can be seen, both on the surface and in the bulk (fracture). However, at the surface, these grains are in a highly aggregated state, and the grain boundaries are not explicit. Nevertheless, in the GIG sample, whose morphology is represented in Figure 4d, there are grains with sizes ranging from 400 nm to more than 1 µm, with the grain boundaries clearly visible. The bulk has an identical morphology, as do the other samples. It should be noted that these extreme compositions showed some porosity, which is no longer visible in the intermediate samples. Figure 4c presents the micrograph of the 75GIG_BTO sample, showing a clear phase separation, with the mixture of the two types of grains distinct both in shape and size. The smaller ones are associated with the barium-containing phase and the larger grains with the gadolinium-containing phase. ImageJ software was used to analyze the scanning electron microscopy data.

### 3.4. Electrical Analysis

Impedance spectroscopy allows us to investigate the fundamental aspects of material properties, yielding a wealth of information about molecular motions. In the case of composites, this technique is particularly useful and frequently complemented by other measurement methods, such as those presented in this work. Furthermore, we calculated the complex permittivity, $\varepsilon^{*}=\varepsilon \prime -i\varepsilon \prime \prime$. The real part, *ε′*, is related to the energy stored in the material, and the imaginary part, *ε″*, is related to the energy dissipated per cycle.

Then, the real, ε′, and the imaginary, ε″, parts of the complex permittivity were calculated from the dielectric measurements [24], as the samples have a size that allows us to consider a parallel plate capacitor:

(4) $$\varepsilon \prime =C_{p}\frac{d}{A\varepsilon_{0}}$$

(5) $$\varepsilon \prime \prime =\frac{d}{\omega R_{p}A\varepsilon_{0}}$$

where *d* represents the sample thickness, *A* the electrode area, *ε_0_* the empty space permittivity and *ω* the angular frequency, respectively. *C_p_* and *R_p_* are the capacitance and resistance in the parallel model configuration.

**Figure 5:**
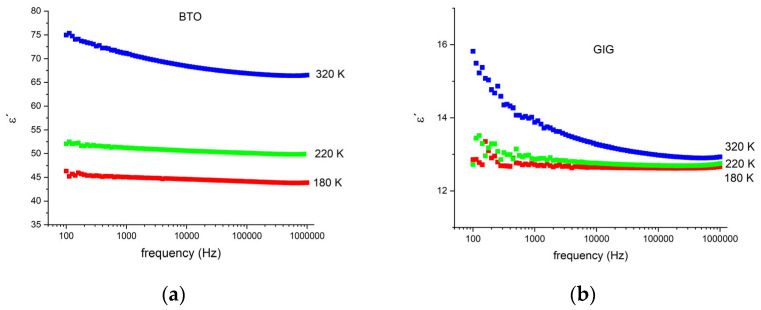
Real part of the complex permittivity, *ε′*, for 3 different temperatures, as a function of frequency for (**a**) BTO and (**b**) GIG.

Figure 5 shows the real part of the complex permittivity, *ε′*, as a function of frequency for the BTO and GIG samples for different temperatures.

As observed, the values increase with temperature and are higher for BTO. The decrease in the dielectric constant, *ε′*, with the frequency occurs as expected and becomes more pronounced with the increase in the temperature. In the low-frequency region, when a time-varying electric field is applied, the polarization can follow the changing electric field, leading to the highest dielectric constant. With the increase in the frequency, the dipolar polarization is not able to keep the alignment with the electric field, leading to a decrease in the total polarization and consequently, a decrease in ε′.

**Figure 6:**
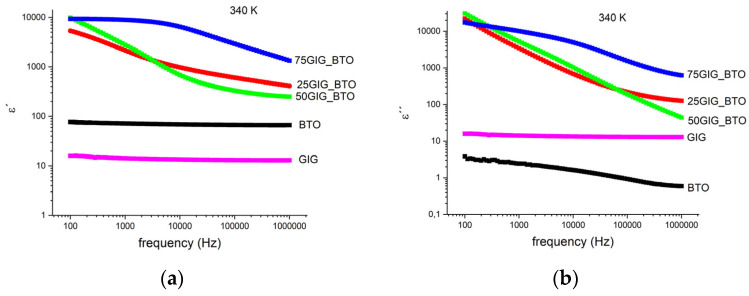
Complex permittivity for all the samples at T = 340 K, as a function of frequency for (**a**) *ε′*: (**b**) ε″.

Figure 6 shows the real and imaginary parts of the complex permittivity, as a function of frequency, at a constant temperature. It is clear that the composites with the BTO and GIG present higher values for the complex permittivity than those of the initial materials. This can be explained by the formation of new phases and eventually the change in the morphology, as observed in the X-ray and SEM analyses.

**Figure 7:**
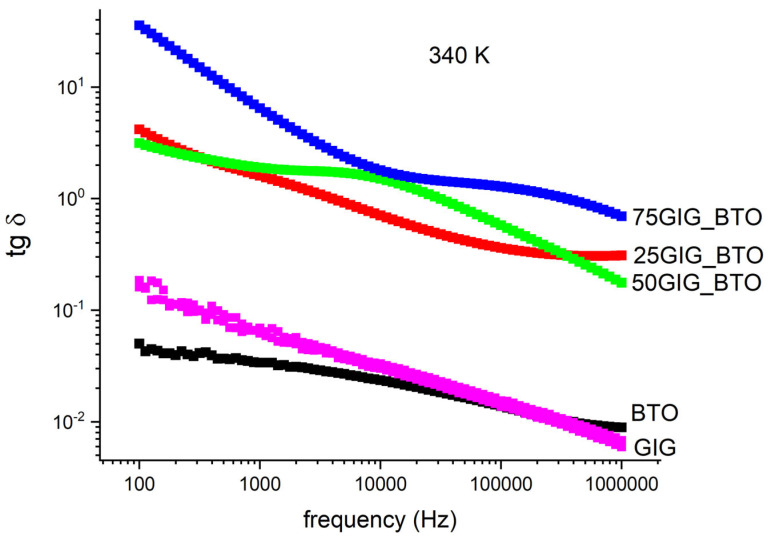
tg δ, as a function of frequency for all the samples at a constant temperature of T = 340 K.

In electronic applications, namely energy storage, for the best balance between the dielectric constant, *ε′*, and the losses, *ε″*, $tg\;\delta =\frac{\varepsilon \prime \prime }{\varepsilon \prime }$ must be considered. This is presented in Figure 7 for a particular constant temperature (340 K).

**Figure 8:**
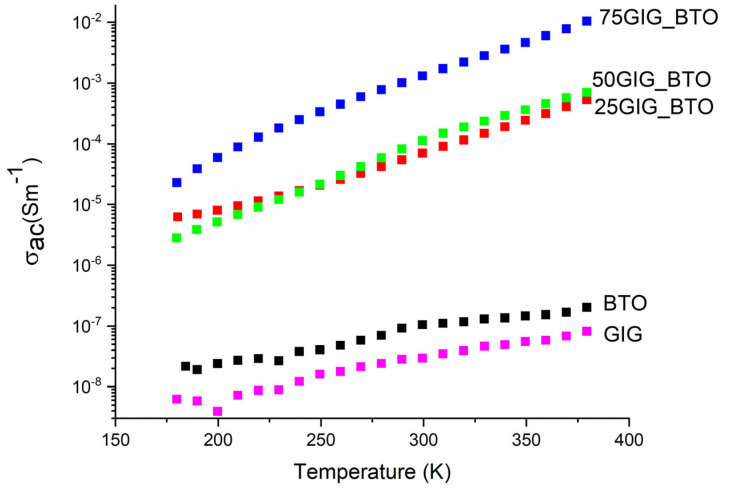
Ac conductivity for all the samples at f = 1 kHz, as a function of temperature.

It can be seen that the 75GIG_BTO sample, which has a high dielectric constant, also presents huge losses, and this is confirmed by the tangent δ. This behavior is obviously undesirable, and thus this composite is not adequate for energy storage systems. Actually, the 25GIG_BTO sample presents the best results and could be used for that purpose. The balance between the dielectric constant and losses is the best for this sample, out of all of the samples. In Figure 8, the ac conductivity is shown at a frequency of 1 kHz, which confirms this conclusion. It is observed that the conductivity for this sample is about 10^−5^ Sm^−1^, which is acceptable for energy storage systems.

## 4. Discussion

The BTO and GIG samples have the lowest values for the complex permittivity, that is, the real and imaginary parts. The formation of a composite starting from these materials leads to a clear increase in the real and imaginary parts of the complex permittivity. Nevertheless, a balance of these two entities is crucial to obtaining a good material for usage in energy storage. The appearance of new phases is responsible for the increase in the dielectric properties, in particular the BaFe_12_O_19_ phase. This behavior has already been observed in several works of other authors. Additionally, some morphological modifications due to the increase in the surface areas of the grains can explain this increase. It was observed that the BTO and GIG compositions showed some porosity, which is no longer visible in the intermediate composite samples. The huge increase in the conductivity for the 75GIG_BTO sample, which also has a high dielectric constant, hinders the use of this composite for energy storage. So, we can infer that the 25GIG_BTO sample can be used for that application. Table 1 shows the calculated values of the dielectric constant and losses at 1 kHz and 300 K. For comparison, other materials are presented with dielectric constants at room temperature and 1 kHz, such as 3.7–10 in glasses, 83–183 in titanium oxide and 310 in strontium titanate [25]. It is observed that the combination of different compounds that have excellent electronic properties leads to new composite materials, as has also been confirmed in other works [26,27].

The electrical properties of the composites were investigated in this work, but, in the future, their magnetic properties should be investigated, as ferrites are promising materials for magnetic storing energy. In particular, they are useful for applications that need rapid bursts of energy, overcoming the existing challenge of low energy density in magnetic systems.

## 5. Conclusions

The dielectric properties of barium titanate/gadolinium ferrite ceramic composites were investigated. Our main objective was to obtain a material with dielectric properties that are good for energy storage, that is, a high dielectric constant and low losses. We used a specific procedure to synthesize the materials which enhanced the electrical properties. A correlation between the morphological, structural and dielectric properties was defined, and the best compromise between the partial compositions (25GIG_BTO) was obtained. A material that could potentially be used in energy storage applications was developed, with the required balance between the dielectric constant and dielectric losses.

## Figures and Tables

**Table 1:** *ε′* and *ε″* for the different samples at T = 300 K and f = 1 kHz.

Sample	ε′	ε″
**BTO**	70	1.9
**25GIG_BTO**	1380	1270
**50GIG_BTO**	1100	2020
**75GIG_BTO**	6800	23,700
**GIG**	14	0.6
